# Efficient Removal of Pb(II) Ions from Aqueous Solutions Using an HFO-PVDF Composite Adsorption Membrane

**DOI:** 10.3390/membranes15090264

**Published:** 2025-09-01

**Authors:** Shuhang Lu, Qianhui Xu, Mei-Ling Liu, Dong Zou, Guangze Nie

**Affiliations:** 1 School of Environmental Science and Engineering, Nanjing Tech University, Nanjing 211816, China; 2 NJTECH University Suzhou Future Membrane Technology Innovation Center, Suzhou 215333, China

**Keywords:** hydrous ferric oxide, composite adsorption membrane, lead removal

## Abstract

The efficient purification of Pb(II)-containing wastewater is essential for safeguarding public health and maintaining the aquatic environment. In this study, novel hydrous ferric oxide (HFO) nanoparticle-embedded poly(vinylidene fluoride) (PVDF) composite adsorption membranes were developed through a simple blending method for efficient Pb(II) removal. This composite membrane (denoted as HFO-PVDF) combines the excellent selectivity of HFO nanoparticles for Pb(II) with the membrane’s advantage of easy scalability. The optimized HFO-PVDF_(1.5)_ membrane achieved adsorption equilibrium within 20 h and exhibited excellent adsorption capacity. Moreover, adsorption capacity markedly enhanced with increasing temperature, confirming the endothermic nature of the process. The developed HFO-PVDF membranes demonstrate significant potential for real-world wastewater treatment applications, exhibiting exceptional selectivity for Pb(II) in complex ionic matrices and could be effectively regenerated via a relatively straightforward process. Furthermore, filtration and dynamic regeneration tests demonstrated that at an initial Pb(II) concentration of 5 mg/L, the membrane operated continuously for 10–13 h before regeneration, treating up to 200 L/m^2^ of wastewater before breakthrough, highlighting potential for cost-effective industrial wastewater treatment. This study not only demonstrates the high efficiency of the HFO-PVDF membrane for heavy metal ion removal but also provides a theoretical foundation and technical support for its practical application in water treatment.

## 1. Introduction

Heavy metal pollutants have garnered significant attention because of their environmental persistence and bioaccumulative characteristics, with lead (Pb) pollution being particularly prominent [1,2]. The toxic effects of lead on the human body primarily involve disrupting the metabolic processes of essential metal elements, leading to toxic effects on multiple organs, i.e., the nervous system, kidneys, and reproductive system [3,4]. Given the severe health risks associated with lead pollution, various countries and regions have established strict drinking water quality standards. The United States Environmental Protection Agency (USEPA) stipulates that the concentration of lead in drinking water must not exceed 0.015 mg/L, whereas the World Health Organization (WHO) and China’s “Sanitary Standard for Drinking Water” (GB5749-2022) require lead levels to remain below 0.01 mg/L [5,6]. Therefore, developing efficient and cost-effective lead pollution control technologies holds significant practical implications for safeguarding public health and maintaining ecological environmental integrity. Currently, various methods such as chemical precipitation, biological treatment, ion exchange, and adsorption are commonly employed for lead removal [7,8,9,10]. However, the widespread application of these methods is often hindered by high costs, complex operations, and challenges in meeting increasingly stringent emission standards. In contrast, adsorption has emerged as an appealing option due to its high removal efficiency, simple operation, and low cost [1,11]. Despite recent advancements in adsorbent materials such as natural clays, carbon materials, and metal oxides for lead removal, their limited adsorption capacities combined with difficulties in elution, reusability, and resource recovery remain major concerns [12].

In recent decades, nanomaterials have garnered significant attention in the field of water treatment due to their large surface area and robust chemical reactivity [13]. Among these materials, nano-metal oxides such as nanosized hydrated zirconium oxide (HZO) [6], hydrated manganese oxide (HMO) [14], and hydrous ferric oxide (HFO) [15] have demonstrated efficient heavy metal removal capabilities owing to their strong inner-sphere complexation and abundant active adsorption sites. However, direct utilization of these nanoparticles is impractical due to potential issues such as self-aggregation, difficulties in separation for recycling, and high pressure drop. To address these challenges, one of the most common approaches is to disperse the nanoparticles onto larger porous carriers [16,17]. Among various carrier materials, membranes are considered ideal carrier materials because of their ability to be easily scalable for various applications [18]. In recent years, significant progress has been made in the fabrication of composite adsorption membranes for heavy metal removal. This approach preserves the favorable mechanical properties of the polymer substrate while successfully introducing the active sites of the nanomaterial into the membrane matrix, resulting in a synergistic enhancement of membrane performance [19,20,21]. Among the various techniques of membrane fabrication, the blending method, which involves the direct doping of nanoparticles into the polymer solution, has garnered considerable attention due to its simple process, ease of operation, and excellent outcomes. This technique involves the uniform dispersion of nanoparticles within the polymer solution, followed by membrane formation processes such as phase inversion to produce functional composite membranes.

In this work, HFO nanoparticles were incorporated into a polyvinylidene fluoride (PVDF) casting solution by blending modification technology. PVDF is selected as the carrier material because this polymer not only exhibits excellent chemical stability and thermal stability but also enables the fabrication of diverse configurations such as flat sheets, hollow fibers, or tubular membranes [22,23], and its solubility characteristics allow it to be processed via mature techniques such as the nonsolvent-induced phase separation (NIPS) method, with relatively low raw material costs [24]. Moreover, the mesh-like polymer chains within PVDF can serve as protective enclosures for the embedded nanoparticles, preventing their aggregation and minimizing their release into the water system. The adsorption performance of the membranes in the treatment of Pb(II)-containing wastewaters, such as the pH, adsorption kinetics, adsorption isotherms, and presence of coexisting ions, was investigated in detail. Our study demonstrated that the HFO-PVDF adsorption membrane exhibited promising potential for lead removal from water. This work provides new insights for the development and application of novel membrane materials.

## 2. Materials and Methods

### 2.1. Materials

Polyvinylidene fluoride (PVDF) powder (Solef 6010, Mw: ~573,000) was obtained from Solvay and dried at 333 K for at least 4 h to eliminate the absorbed water. Fe(NO_3_)_3_·9H_2_O, polyvinylpyrrolidone (PVP, K30), NaCl, Na_2_SO_4_, NaNO_3_, NaOH, HCl, and Pb(NO_3_)_2_ were obtained from Sinopharm Chemical Reagent Co., Ltd. (Beijing, China). N,N-Dimethylformamide (DMF) was purchased from Aladdin Company. The reagents were used without further purification. A stock solution of 1000 mg∙L^−1^ Pb was prepared by dissolving Pb(NO_3_)_2_ in deionized water. Deionized water was used throughout the entire experiment.

### 2.2. Fabrication of HFO-PVDF Membranes

HFO nanoparticles were synthesized via a precipitation method. First, 60 g of Fe(NO_3_)_3_·9H_2_O was dissolved in 750 mL of deionized water. Next, 1 mol/L NaOH was added dropwise until the pH reached 7. The suspension was maintained at pH 7 for 72 h. After the reaction, the mixture was centrifuged, and the precipitate was washed with deionized water 5–6 times to remove excess ions. Finally, the pulverized HFO was obtained after freeze-drying.

HFO-PVDF adsorption membranes were fabricated via nonsolvent-induced phase separation. A defined mass of HFO nanoparticles was ultrasonically dispersed in 30 mL of DMF. After vigorous stirring for 1 h, 1.5 g of PVP and 4.0 g of PVDF were introduced, followed by continuous stirring at 70 °C for 6 h to obtain a homogeneous HFO-incorporated PVDF casting solution. The solutions were first degassed ultrasonically to eliminate air bubbles and then cast onto a clean glass substrate via a 200-μm doctor blade. Following a 30 s exposure to ambient atmosphere, the cast membrane was immersed in a water coagulation bath for phase inversion. The composite adsorption membranes, labeled HFO-PVDF, were dried to a constant weight in a 50 °C vacuum oven. Pure PVDF membranes were synthesized identically without the addition of HFO.

### 2.3. Analysis and Characterization

The concentration of Pb in the solution sample was quantified via inductively coupled plasma (ICP) emission spectroscopy (ICP-OES, ICP-5000, Focused Photonics Inc., Hangzhou, China). Following digestion with HNO_3_-HClO_4_, the iron content in the HFO-PVDF membrane was quantified. The morphologies of the samples were examined by scanning electron microscopy (SEM, ZEISS Sigma 360, Oberkochen, Germany). The pore size distribution and specific surface area were evaluated via nitrogen adsorption–desorption measurements conducted at 77 K (ASAP 2460, Micromeritics, Norcross, GA, USA). The characteristic groups and crystal structures of the membrane materials were characterized via Fourier transform infrared (FTIR) spectroscopy (Thermo Nicolet8700, Thermo, Waltham, MA, USA) and X-ray diffraction (XRD, Rigaku MiniFlex600, Rigaku, Tokyo, Japan). The changes in the material before and after Pb(II) adsorption were investigated via X-ray photoelectron spectroscopy (XPS) via a Thermo Fisher Nexsa instrument with an Al Kα source, and the possible adsorption mechanism was verified. Contact angle measurements were conducted via a contact angle instrument (OCA20, datatorics, Filderstadt, Germany). The pure water flux of the membrane was measured via a cross-flow cylindrical UF stirred cell. The membrane sample was first pressurized at 2 bar for 30 min to avoid any compaction effects, and the permeation experiment was subsequently carried out at 1 bar. The pure water flux of the membranes was calculated via Equation (1), written as follows:

J_w_ = Q/AΔt (1)

where J_w_ is the pure water flux (L/m^2^·h), Q is the volume of collected permeate (L), A is effective membrane area (m^2^), and t is the sampling time (h).

### 2.4. Batch Adsorption Experiments

Batch adsorption experiments were performed by adding 25 mg of material to 50 mL of solution in 150 mL conical flasks, which were agitated at 140 rpm for 24 h in a constant-temperature shaker. These experiments aimed to evaluate the Pb adsorption efficiency of HFO-PVDF membranes under various conditions, including initial Pb concentrations, pH levels, contact times, and the presence of coexisting ions. The accuracy of the results was ensured through the implementation of three replicates. The pH of the Pb(II)-containing solution was adjusted using 0.1 M HCl and 0.1 M NaOH solutions. The kinetic test was performed at various time intervals. NaCl, Na_2_SO_4_, and NaNO_3_ were selected as sources of coexisting anions. To evaluate the recycling performance and stability, an HCl solution (pH = 3) was used as the regenerator and used to rinse the depleted adsorbent for the next adsorption run.

### 2.5. Membrane Filtration Experiments

Filtration experiments were carried out in a 50 mL ultrafiltration cup with HFO-PVDF membranes at 298 K. The volume flow was controlled by a variable speed peristaltic pump (Langer, BQ50-1 J, Baoding, China), and the effluent samples were collected by an automatic fraction collector. The effluent was collected at different intervals to measure the residual Pb(II) concentration. After the completion of the filtration experiment, the membrane was regenerated. Subsequently, filtration tests were performed on the regenerated membrane under the same conditions. The permeation flux of the membrane was calculated via Equation (2), written as follows:

F = V/(A × t)(2)

where F is flux (L·m^2^·h^−^^1^), V is the permeate water volume (L), A is the effective membrane area (m^2^), and t is time of collecting permeate water (h).

## 3. Results and Discussion

### 3.1. Preparation and Characterization of the HFO-PVDF Membranes

**Figure 1:**
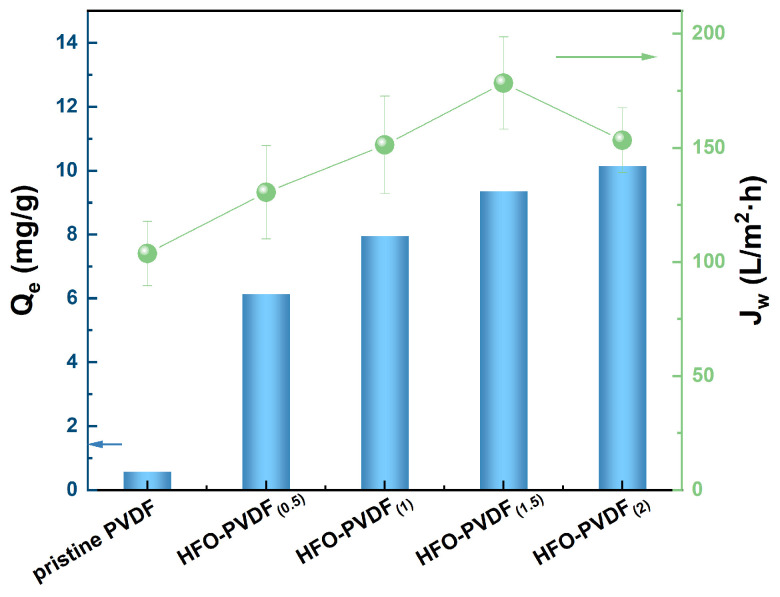
Adsorption of Pb(II) and water flux characteristics of the synthesized membranes.

To optimize membrane fabrication, the effects of the HFO addition on the properties of the adsorption membranes were investigated. A series of HFO-PVDF composite adsorption membranes, denoted as HFO-PVDF_(i)_, where *i* represents the HFO-to-PVDF mass ratio in the casting solution (ranging from 0.5–2), were fabricated, with a pristine PVDF membrane serving as the control. As shown in Figure 1, the Pb(II) removal efficiency of the composite membranes increased with increasing HFO content. This improvement is attributed primarily to the increased number of active sites provided by HFO. However, the HFO-PVDF_(1.5)_ membrane presented the highest pure water flux (178.35 L·m^−2^·h^−1^). In contrast, although the HFO-PVDF_(2)_ membrane contained a greater proportion of the active component and possessed greater adsorption capacity, its flux decreased to 153.28 L·m^−2^·h^−1^. This indicates that excessive HFO loading may reduce membrane porosity and promote particle agglomeration, adversely affecting water permeance and potentially hindering further improvement in adsorption performance. Therefore, HFO-PVDF_(1.5)_ was selected as the optimized membrane for subsequent Pb(II) removal.

**Figure 2:**
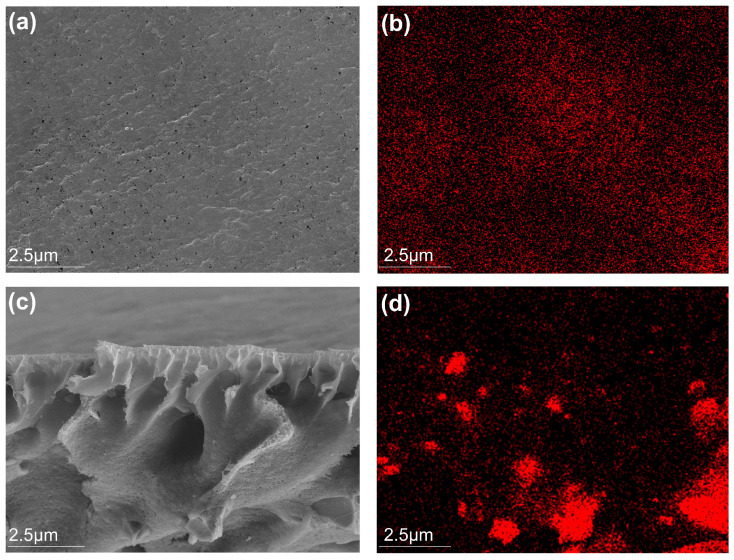
SEM and Fe elemental mapping images of the HFO-PVDF membrane: (**a**,**b**) surface images, (**c**,**d**) cross-sectional images.

**Figure 3:**
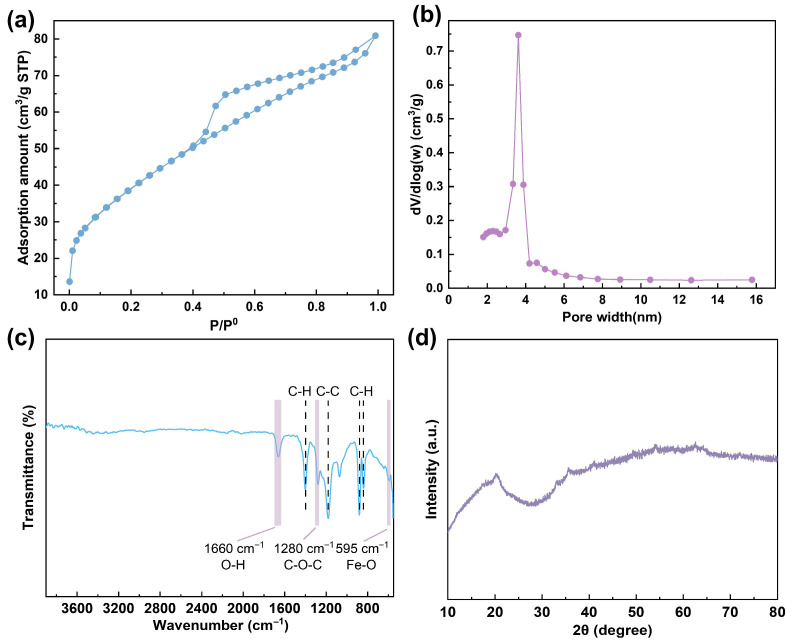
Microstructure characterization of the HFO-PVDF membrane: (**a**) N_2_ adsorption isotherms, (**b**) pore size distribution based on the BJH model, (**c**) FTIR spectra, and (**d**) XRD spectra.

SEM images of the HFO-PVDF_(1.5)_ membrane are presented in Figure 2. The membrane surface was defect-free and exhibited well-defined porous structures. The cross-sectional view revealed an interconnected pore architecture characterized by finger-like macrovoids transitioning into spongy sublayers. SEM-EDX elemental mapping demonstrated the homogeneous distribution of iron (Fe) throughout both the membrane surface and cross-section, indicating uniform HFO loading within the PVDF matrix. The specific surface area and pore structure of the composite membranes were analyzed using nitrogen adsorption–desorption measurements. As shown in Figure 3a, the adsorption isotherm of the HFO-PVDF membrane exhibited a Type-IV pattern with an H4-type hysteresis loop, suggesting the presence of mesopores and narrow slit-shaped pores. The Barrett–Joyner–Halenda (BJH) model revealed a mean pore size of 3.6 nm (Figure 3b), a dimension conducive to rapid ion diffusion and enhanced accessibility to the HFO nanoparticles. Furthermore, Brunauer–Emmett–Teller (BET) analysis indicated a specific surface area of 141.66 m^2^·g^−1^, providing a substantial number of active sites for Pb(II) binding. FTIR spectra (Figure 3c) clearly showed characteristic peaks corresponding to the PVDF polymer and Fe-O vibrations, further confirming the successful incorporation of HFO into the PVDF membrane matrix. The XRD patterns in Figure 3d revealed no characteristic peaks of crystalline iron oxides, indicating that the loaded iron oxides were predominantly amorphous HFO.

### 3.2. Effect of pH on Pb(II) Removal

**Figure 4:**
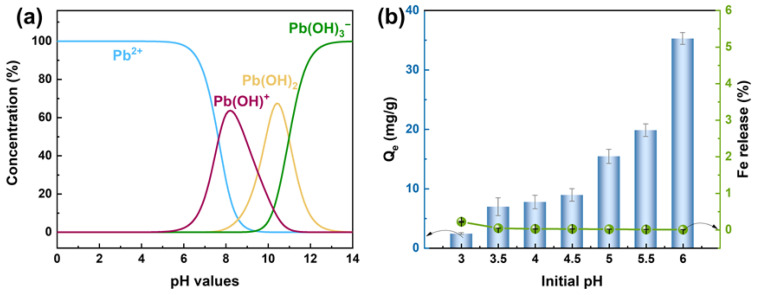
(**a**) Species distribution of lead in aqueous solution, (**b**) effect of pH on Pb(II) adsorption and Fe leaching.

The pH of the solution is a critical factor influencing the adsorption process, as it governs the surface charge properties of the adsorbent, the speciation of the adsorbate, and its solubility. Considering that Pb(II) ions precipitate at pH values exceeding 6 under the experimental concentration (25 mg/L), the adsorption behavior of Pb(II) onto the HFO-PVDF membrane was investigated within the pH range of 3–6. The Pb(II) species distribution curve at different pH values in Figure 4a indicates that lead predominantly exists as Pb^2+^ ions within the pH of 3–6. This suggested that Pb(II) removal within this pH interval primarily occurs through adsorption rather than precipitation.

The effect of pH on the Pb(II) adsorption performance of the HFO-PVDF membrane is presented in Figure 4b. The adsorption capacity significantly increased with increasing pH, increasing from 2.5 mg/g to ~35 mg/g. This phenomenon can be attributed to two primary factors: (1) In acidic environments, high concentrations of H^+^ ions compete with Pb(II) ions for active sites on the adsorbent surface, reducing Pb(II) uptake; (2) Under low pH conditions, the material surface undergoes protonation, acquiring a positive charge. This resulted in electrostatic repulsion against the positively charged Pb(II) ions. These dual effects collectively suppressed the adsorption process. As the pH increased, the decreased H^+^ concentration led to surface deprotonation, resulting in the surface charge properties. This weakened the electrostatic repulsion and facilitated the approach and occupation of adsorption sites by Pb(II) ions, thereby increasing the adsorption efficiency.

Moreover, the leaching behavior of HFO from the composite membrane under various pH conditions was evaluated, as shown in Figure 4b. The results demonstrated negligible Fe leaching from the HFO-PVDF membrane within the pH range of 3–6, indicating its excellent chemical stability. This stability was due primarily to the acid resistance of PVDF polymers. The combination of this acid resistance with the efficient adsorption capability of HFO rendered the HFO-PVDF adsorption membrane advantageous for treating acidic wastewater, particularly demonstrating outstanding performance in removing heavy metal ions.

### 3.3. Adsorption Kinetics

**Figure 5:**
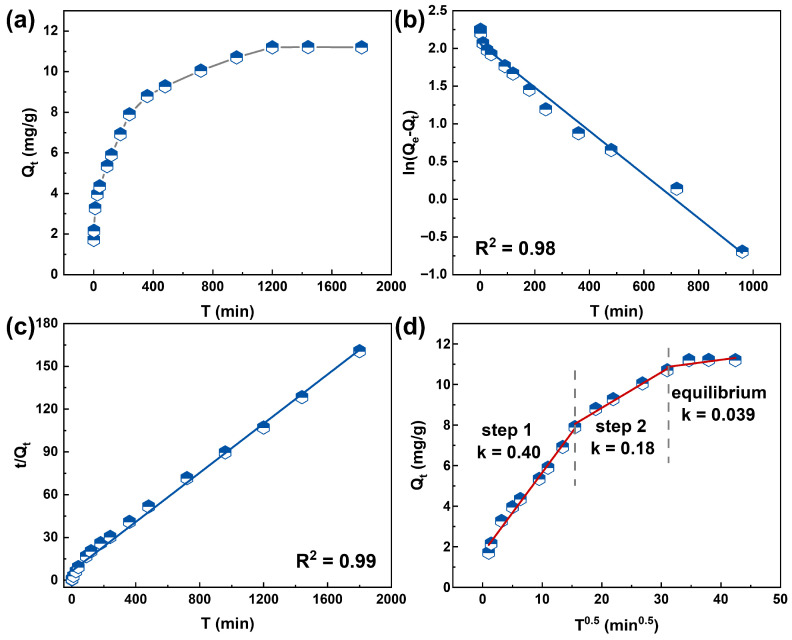
(**a**) Adsorption kinetics of Pb(II) on the HFO-PVDF membrane, and the fitting results of the (**b**) pseudo-first-order model, (**c**) pseudo-second-order model, and (**d**) intraparticle diffusion model.

Adsorption kinetic experiments were conducted to investigate the adsorption efficiency of the HFO-PVDF membrane for Pb(II) and the evolution of its adsorption capacity as a function of time. As shown in Figure 5a, the adsorption process on the HFO-PVDF membrane exhibited distinct stages. A rapid adsorption phase was characterized during the initial 200 min, followed by a gradual decline in the adsorption rate and ultimately reached equilibrium at 1200 min. This dynamic profile intuitively reflected the progressive saturation of the active sites on the membrane surface.

Furthermore, pseudo-first-order and pseudo-second-order kinetic models were employed to analyze the adsorption behavior of Pb(II) onto the HFO-PVDF membrane. The fitting results are presented in Figure 5b,c. The equilibrium adsorption capacities (Q_e_) were 10.42 mg/g (pseudo-first-order model) and 11.31 mg/g (pseudo-second-order model) for Pb(II). The fitting results indicated that the pseudo-second-order kinetic model (R^2^ = 0.99) provided a more accurate description of the Pb(II) adsorption process. Furthermore, the Q_e_ value calculated from the pseudo-second-order model was consistent with the experimental results.

To gain deeper insights into the adsorption kinetics mechanism, the intraparticle diffusion model was also applied. The core equation of this model is the Weber-Morris equation, expressed as follows:

(3) $$Q_{t}\;=\;kt^{\;\frac{1}{2}}\;+\;C$$

where *Q_t_* is the adsorption capacity at time *t* (mg·g^−1^), *k* is the intraparticle diffusion rate constant (mg·g^−1^·min^1/2^), and C is a constant related to the boundary layer thickness (mg·g^−1^).

As shown in Figure 5d, the adsorption process of Pb(II) onto the HFO-PVDF composite membrane can be divided into three dynamic stages with the diffusion rate decreasing progressively throughout the process, which reveals the rate-controlling mechanisms during different adsorption stages. In the initial stage, the adsorption membrane exhibited rapid adsorption, which was primarily controlled by external diffusion. The large concentration gradient between the bulk solution and the adsorbent surface resulted in the minimal mass transfer resistance, facilitating the adsorption of Pb(II) onto the membrane surface. The steep slope of the fitted line indicates a high adsorption rate. As adsorption proceeded, active sites on the HFO surface became progressively occupied, and Pb(II) began diffusing into the interior of the HFO particles. Compared with that of external diffusion, the diffusion rate during this stage significantly decreased, as Pb(II) had to overcome the mass transfer resistance within the internal pore channels of the membranes. Consequently, the adsorption rate gradually slowed, as reflected by a decrease in the slope of the fitted line. When the active sites on both the surface and interior of HFO approached saturation, the adsorption process reached a dynamic equilibrium plateau. Notably, all fitted lines exhibited a nonzero intercept, indicating that the adsorption process was not governed solely by a single diffusion mechanism but rather resulted from the combined effects of both external diffusion and intraparticle diffusion [25].

### 3.4. Adsorption Isotherm

**Figure 6:**
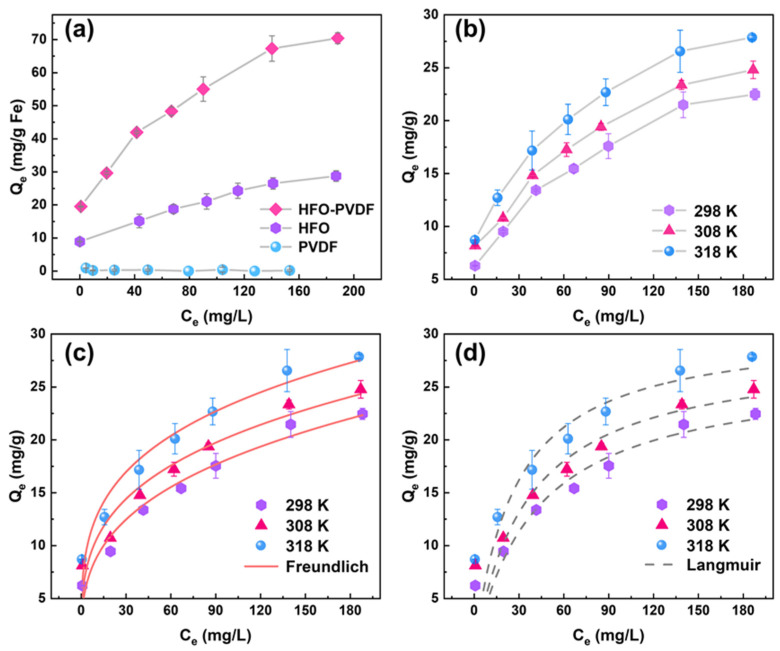
(**a**) Adsorption of Pb(II) on the PVDF membrane, HFO powder, and HFO-PVDF membrane; (**b**) adsorption isotherms of Pb(II) on the HFO-PVDF membrane; and the fitting curves of the (**c**) Langmuir and (**d**) Freundlich models at different temperatures.

Adsorption isotherm experiments were conducted to evaluate the adsorption performance of the HFO-PVDF membrane for Pb(II), and its adsorption capacity was compared with that of both the pristine PVDF membrane and HFO powders. The experimental results, presented in Figure 6a, demonstrated that the PVDF membrane exhibited negligible adsorption capacity for Pb(II). In contrast, the HFO-PVDF membrane displayed a significantly higher Pb(II) adsorption capacity. This enhancement in adsorption performance was attributed to the incorporation of HFO nanoparticles within the membrane matrix. In addition, the HFO component within the composite membrane exhibited greater adsorption efficiency compared to pure HFO powder. Specifically, the Pb(II) adsorption capacity per unit mass of Fe in the HFO-PVDF membrane was notably greater than that of the HFO powders. This occurred because the HFO nanoparticles in their pure powders were prone to agglomeration due to van der Waals forces among the particles, which reduced the specific surface area and decreased the number of accessible active sites. Conversely, in the HFO-PVDF composite membrane, the PVDF matrix effectively suppressed the agglomeration of HFO nanoparticles and thus exposed a greater number of accessible adsorption sites, significantly enhancing the Pb(II) adsorption capacity [26].

The influence of temperature on Pb(II) adsorption by the HFO-PVDF membrane was further investigated, as depicted in Figure 6b. The adsorption capacity of Pb(II) onto the membrane steadily increased as the temperature increased from 298 K to 318 K. This phenomenon suggested that the adsorption process was endothermic, and increasing the temperature favored Pb(II) adsorption by the HFO-PVDF membrane. To gain deeper insights into the adsorption behavior, the isotherm data for Pb(II) adsorption onto the HFO-PVDF membrane were fitted using the Langmuir and Freundlich models. The fitting results are presented in Figure 6c,d, with the corresponding fitting parameters summarized in Table 1. Across all three temperatures, the Freundlich model yielded higher correlation coefficients (R^2^) than the Langmuir model, indicating its superior ability to accurately describe the Pb(II) adsorption behavior and suggesting that the adsorption process tended toward a multilayer chemisorption mechanism. Furthermore, the parameter K_F_ in the Freundlich model was closely associated with the adsorption capacity of the adsorbent. A higher K_F_ value signifies a greater affinity for the adsorbate, implying a more favorable adsorption process. As shown in Table 1, the K_F_ value at 318 K was greater than those at 308 K and 298 K. This observation provided additional confirmation that an increase in temperature facilitated the adsorption of Pb(II) onto the HFO-PVDF membrane.

The thermodynamic parameters for the adsorption of Pb(II) from aqueous solution onto the composite membrane were calculated via the Van’t Hoff equation:

(4) $$\Delta G^{0}\;=\;-\mathrm{RTln}K_{0}$$

(5) $$\ln K_{0}=\frac{\Delta S^{0}}{R}-\frac{{\Delta H}^{0}}{\mathrm{RT}}$$

where ΔG^0^ is the standard Gibbs free energy change (J·mol^−1^), T is the absolute temperature (K), R is the ideal gas constant (8.314 J·mol^−1^·K^−1^), K_0_ is the thermodynamic equilibrium constant (Q_e_/C_e_), ΔH^0^ is the standard enthalpy change (J·mol^−1^), and ΔS^0^ is the standard entropy change (J·mol^−1^·K^−1^).

**Figure 7:**
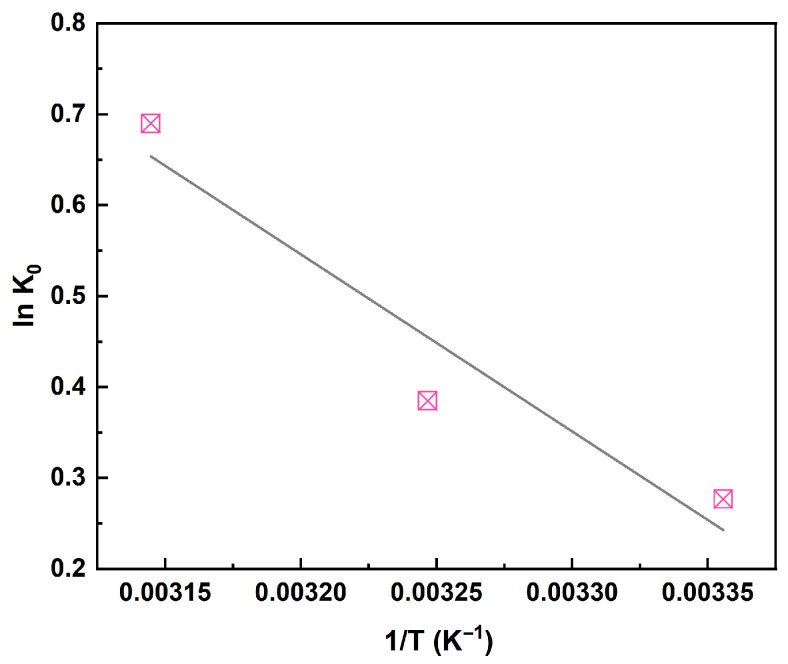
Linear relationship between ln K_0_ and 1/T.

The linear relationship between ln K_0_ and 1/T was analyzed (Figure 7). The slope of the linear plot was −ΔH^0^/R, and the intercept was ΔS^0^/R. The calculated thermodynamic parameters are listed in Table 2. The ΔG^0^ values were negative and decreased progressively with increasing temperature. This indicated that the adsorption process was thermodynamically spontaneous and became more favorable at elevated temperatures, which was likely attributable to the interaction mechanism between the adsorbent and adsorbate. Furthermore, the ΔH^0^ values were consistently positive. This further confirmed that the adsorption of Pb(II) onto the HFO-PVDF membrane from aqueous solution was an endothermic process. This endothermic nature suggested that the adsorption process likely involved the formation of chemical bonds or interactions with functional groups, leading to stronger chemical bonding between the adsorbent surface and Pb(II) ions, rather than simple physisorption.

### 3.5. Effects of Coexisting Ions

**Figure 8:**
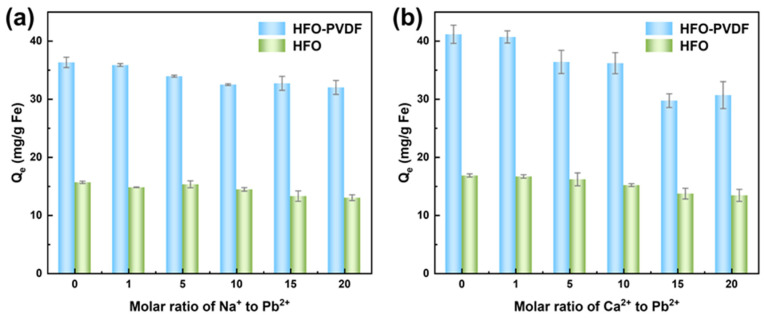
Adsorption of Pb(II) on the HFO-PVDF membrane in the presence of (**a**) Na^+^ and (**b**) Ca^2+^.

In real aqueous environments, target pollutants always coexist with various interfering substances, including inorganic salts, organic matter, and other dissolved species. These interferents compete with the target pollutant for the limited adsorption sites on the adsorbent surface, thereby reducing its removal efficiency. To evaluate the adsorption selectivity of the HFO-PVDF membrane, Na^+^ and Ca^2+^ were selected as representative interfering ions to investigate their impact on the Pb(II) removal efficiency. A systematic experimental analysis was conducted, with HFO powders serving as the control group (both materials have the same iron content). The results are presented in Figure 8.

As the concentration of competing cations increased, only a slight reduction in Pb(II) removal efficiency was observed for both the HFO-PVDF membrane and the HFO powders. Notably, among the two competing cations, divalent Ca^2+^ had a stronger interfering effect on the adsorption process than the monovalent Na^+^. This difference may stem from the smaller ionic radius and higher charge density of Ca^2+^, enabling it to bind more effectively to the active sites on the adsorbent surface and consequently exerting a stronger competitive effect against Pb(II) adsorption. Despite the potential occupation of some adsorption sites by high concentrations of interfering cations, the HFO-PVDF membrane still demonstrated favorable selective adsorption capability. This may be attributed to the ability of Pb(II) ions to form more stable coordination bonds with hydroxyl groups on the HFO surface compared to other cations, thereby maintaining high removal efficiency in complex aqueous environments. Furthermore, the porous structure of the HFO-PVDF membrane and the large specific surface area of HFO provided abundant active sites for Pb(II) adsorption, further enhancing its adsorption performance under competitive conditions.

**Figure 9:**
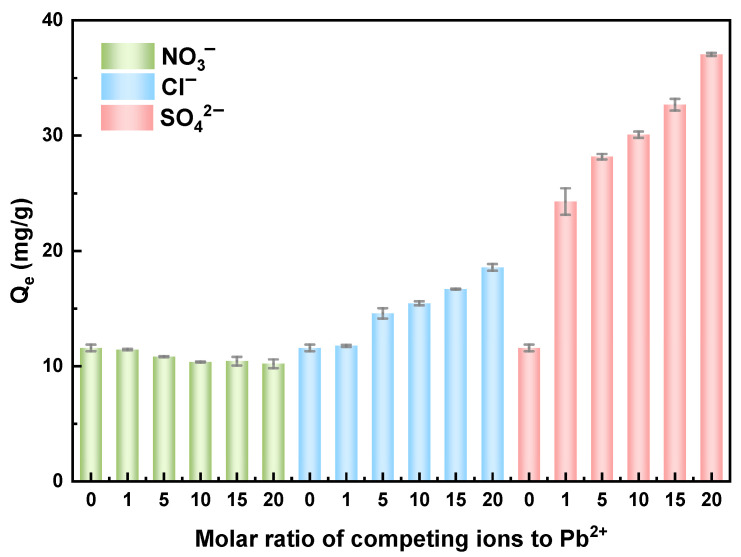
Adsorption of Pb(II) on the HFO-PVDF membrane in the presence of NO_3_^−^, Cl^−^ and SO_4_^2−^.

To comprehensively assess the influence of different ions on the Pb(II) removal efficiency, several common charged anions (including NO_3_^−^, Cl^−^, and SO_4_^2−^) were also selected for analysis. As shown in Figure 9, NO_3_^−^ exhibited almost no influence on Pb(II) adsorption, with the adsorption capacity decreasing only marginally from 11.58 mg/g to 10.20 mg/g when the concentration of these competing anions increased. Paradoxically, the presence of high concentrations of SO_4_^2−^ and Cl^−^ significantly enhanced the Pb(II) removal efficiency of the composite membrane. This phenomenon may originate from the following mechanisms. First, SO_4_^2−^ can undergo a chemical reaction with Pb(II) to form insoluble PbSO_4_ precipitate. Second, SO_4_^2−^ adsorbed onto the adsorbent surface may form the ternary surface complexes, thereby improving the adsorption efficiency [27]. Additionally, high concentrations of Cl^−^ may form stable complexes with Pb(II), such as PbCl_2_. This complexation reduced the concentration of free Pb(II) ions in solution, indirectly augmenting the overall Pb(II) removal capacity. In general, the HFO-PVDF membrane demonstrated robust Pb(II) removal performance in complex, real aqueous environments.

### 3.6. Dynamic Filtration

**Figure 10:**
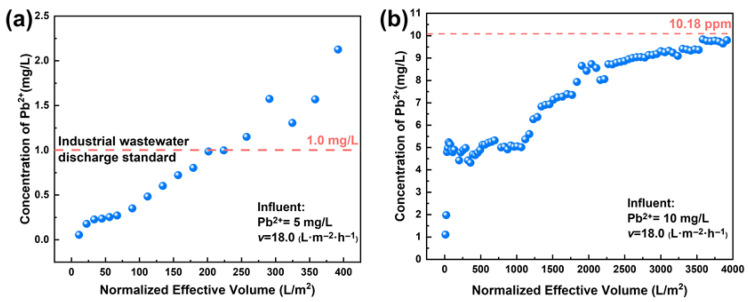
Dynamic filtration of the HFO-PVDF membrane in the presence of (**a**) 5 mg/L and (**b**) 10 mg/L Pb(II).

Simulating the performance evolution of membranes under continuous operation allows for a comprehensive and systematic assessment of their long-term stability and durability. In this work, the Pb(II) concentration specified by the industrial wastewater discharge standard of China was defined as the breakthrough point (1.0 mg/L). As shown in Figure 10a, the HFO-PVDF membrane treated approximately 225 L·m^−2^ of Pb-containing wastewater before reaching the breakthrough point with an influent Pb(II) concentration of 5.0 mg/L. This indicated that the membrane not only exhibited high removal efficiency but also possessed favorable stability when treating low-concentration Pb(II)-containing wastewater.

When the influent Pb(II) concentration was further increased to 10 mg/L, it achieved adsorption saturation only after treatment with 4000 L·m^−2^, although the HFO-PVDF membrane reached the breakthrough point more rapidly (see Figure 10b). This demonstrated that the membrane maintained a high adsorption capacity even when challenged with wastewater containing relatively high concentrations of Pb(II). The dynamic filtration experiments provided critical evidence for evaluating the performance of the HFO-PVDF membrane in treating Pb(II)-containing wastewater at different concentrations. These results further substantiated its substantial application potential in real wastewater treatment.

### 3.7. Antifouling Performance

**Figure 11:**
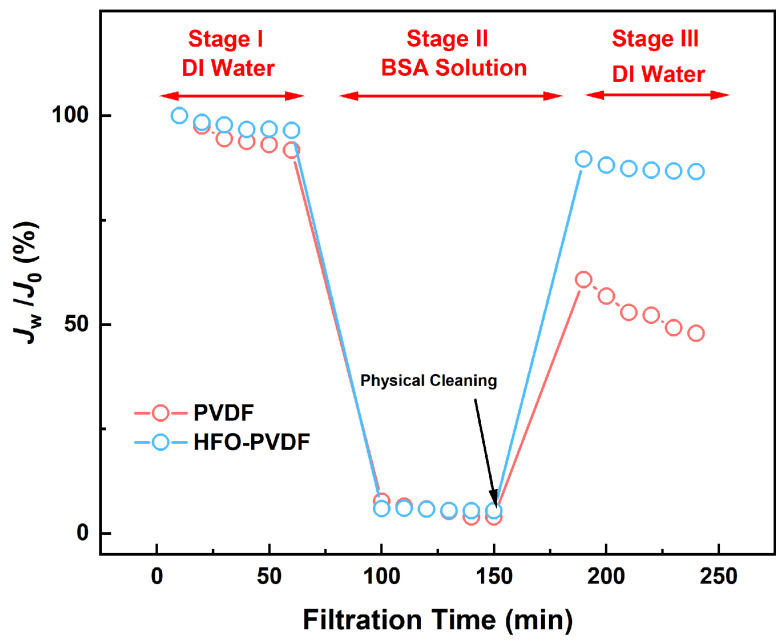
Antifouling properties of the PVDF and HFO-PVDF membranes.

Membrane fouling strongly affects the application performance of the resulting adsorption membrane. In this study, bovine serum albumin (BSA) was used as a model foulant to elucidate the membrane fouling mechanism. As shown in Figure 11, compared with the pure PVDF membrane, the HFO-PVDF adsorption membrane exhibited superior fouling resistance. After a 1 g/L BSA solution was filtered, the HFO-PVDF membrane achieved a flux recovery ratio (FRR) of approximately 90%, whereas the PVDF membrane recovered only 52.19%. This indicated that the HFO-PVDF composite membrane more effectively mitigated foulant adsorption and deposition during filtration, thereby maintaining greater flux stability. Table 3 shows the antifouling indexes of the membranes, including the total fouling ratio (R_t_), the reversible fouling ratio (R_r_), and the irreversible fouling ratio (R_ir_) [28,29]. R_t_ reflects the overall extent of fouling; R_r_ represents the proportion of foulants adsorbed on the surface or causing physical blockage; and R_ir_ represents the proportion of foulants causing pore blockage within the membrane matrix or through chemical adsorption. Compared with the pure PVDF membrane, the HFO-PVDF membrane had a significantly greater Rr of 84.14%. The fouling of the HFO-PVDF membrane predominantly originated from physical deposition on the surface rather than from chemical adsorption. The enhanced antifouling ability of the HFO-PVDF membrane was attributed primarily to its modified properties following blending. The modified composite membrane possessed higher hydrophilicity and lower surface roughness, effectively reducing BSA adsorption and mitigating the fouling rate. Consequently, the HFO-PVDF adsorption membrane maintained stable performance during prolonged operation.

### 3.8. Adsorption Mechanism

**Figure 12:**
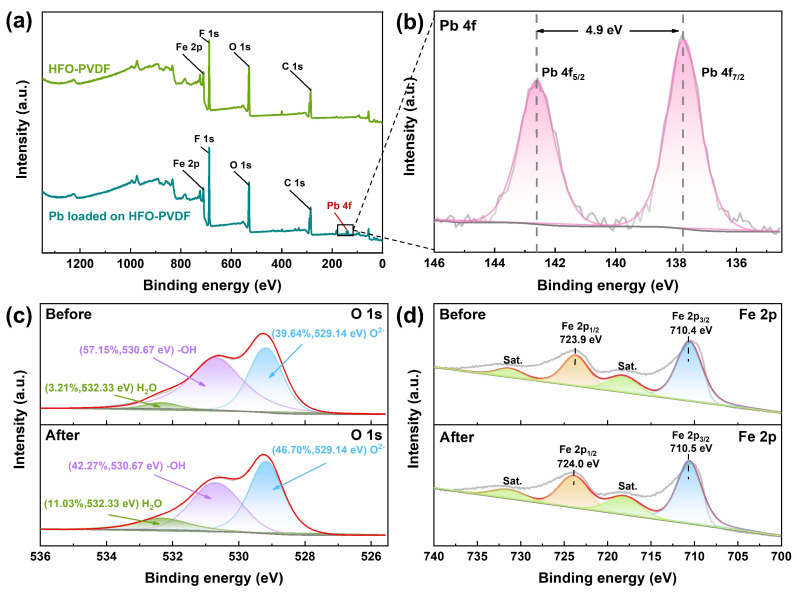
XPS analysis of the HFO-PVDF membrane before and after Pb(II) loading: (**a**) XPS full spectrum; (**b**) Pb 4f; (**c**) O 1s; (**d**) Fe 2p.

X-ray photoelectron spectroscopy (XPS) was employed to investigate the potential adsorption mechanism of Pb(II) onto the HFO-PVDF composite material as presented in Figure 12. The XPS spectrum of the membranes before adsorption (Figure 12a) revealed characteristic peaks for Fe and O, confirming the successful loading of Fe-containing groups within the PVDF membrane. Following Pb(II) adsorption, a distinct Pb 4f peak emerged in the spectrum, indicating the effective immobilization of Pb(II) on the HFO-PVDF membrane. Further analysis of the high-resolution Pb 4f spectrum (Figure 12b) revealed two characteristic peaks at 137.8 eV and 142.7 eV, corresponding to Pb 4f_7_/_2_ and Pb 4f_5_/_2_, respectively [30]. These binding energies were consistent with the reported XPS signatures for Pb(II), confirming the adsorption of Pb(II) onto the membrane. Analysis of the high-resolution O 1s spectrum (Figure 12c) identified different oxygen species present on the membrane surface, including hydroxyl oxygen (-OH), and lattice oxygen (O^2−^), as well as oxygen from molecular water and hydroxides. The relative proportions of these oxygen components were calculated and annotated. A significant decrease in the surface hydroxyl oxygen content from 57.15% to 42.27% was observed after adsorption. This reduction suggested that Pb(II) underwent a complexation reaction with the hydroxyl groups on the HFO surface. Furthermore, analysis of the Fe 2p spectra revealed shifts in the binding energy following Pb(II) adsorption. As shown in Figure 12d, the characteristic peaks located at 723.9 eV and 710.4 eV before adsorption corresponded to Fe 2p_1_/_2_ and Fe 2p_3_/_2_, respectively, confirming the presence of Fe(III) in the composite membrane. After Pb(II) adsorption, both peaks shifted toward lower binding energies. This shift indicated the likely formation of a new Fe-O-Pb coordination structure during adsorption, resulting from interactions between the metal–ligand and the iron (hydr) oxide surface.

### 3.9. Evaluation of Practical Application Potential

**Figure 13:**
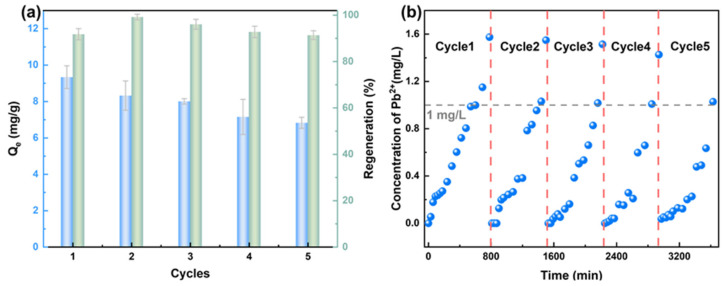
(**a**) Static and (**b**) dynamic regeneration of the HFO-PVDF membranes.

In wastewater treatment, the regeneration capability of an adsorbent is a crucial indicator for assessing its performance stability and economic value. Here, an acidic regeneration method (HCl solution at pH = 3) was employed to investigate the regeneration performance of the HFO-PVDF membrane through multiple static adsorption–desorption cycles and dynamic regeneration experiments. As shown in Figure 13a, the HFO-PVDF membrane maintained good adsorption performance for a 25 mg/L Pb(II) solution at pH = 5 even after five adsorption–desorption cycles and exhibited efficient regeneration with a stable desorption efficiency exceeding 90%. This indicated that the membrane was highly reusable. Furthermore, the HFO-PVDF membrane underwent five consecutive dynamic filtration cycles. Figure 13b shows that the membrane could operate continuously for 10–13 h before regeneration, processing up to 200 L/m^2^ of Pb(II)-containing wastewater at the breakthrough point when the lead concentration was 5 mg/L. These results confirmed that the HFO-PVDF adsorption membrane not only exhibited high-efficiency heavy metal removal but also exhibited excellent reusability.

## 4. Conclusions

In this work, the removal performance of the HFO-PVDF adsorption membrane for Pb(II) was systematically evaluated. The HFO-PVDF membrane exhibited significant adsorption capacity across a wide pH range (pH = 3~6), enabling the efficient removal of Pb(II) ions from aqueous solutions. This demonstrated that the membrane exhibited good adaptability from acidic to weakly alkaline conditions. The isotherm results indicated that the adsorption process was spontaneous and that the adsorption efficiency increased markedly with increasing temperature. This finding revealed the thermodynamic nature of the adsorption process, where elevated temperatures favored enhanced Pb(II) adsorption capacity. This enhancement was likely attributable to intensified interactions between Pb(II) and the membrane surface active sites. The adsorption equilibrium time was approximately 20 h, and the adsorption kinetics were governed by a combination of intraparticle diffusion and external diffusion. In complex ionic environments, the HFO-PVDF membrane maintained strong adsorption selectivity, preferentially adsorbing Pb(II) while exhibiting good tolerance toward interference from other coexisting ions. This high selectivity endows the membrane with a distinct advantage in removing trace heavy metal ions from water. Filtration and cyclic regeneration tests demonstrated that the membrane maintained a stable desorption rate exceeding 90% after five regeneration cycles under static conditions. Furthermore, it exhibited sustained operation for 10–13 h under dynamic regeneration conditions. These attributes suggest its broad application prospects in the water treatment sector, offering a promising and sustainable technological solution for addressing heavy metal pollution challenges.

## Figures and Tables

**Table 1:** Isotherm fitting parameters for Pb(II) adsorption on the HFO-PVDF membrane.

T (K)	Langmuir			Freundlich		
	Q_max_ (mg/g)	K_L_ (L/mg)	R^2^	K_F_ (g/L)	1/n	R^2^
298	26.73	0.02	0.83	4.23	0.04	0.95
308	28.63	0.03	0.75	5.45	0.29	0.93
318	30.66	0.02	0.76	6.88	0.27	0.96

**Table 2:** Thermodynamic parameters of Pb(II) adsorption on the HFO-PVDF membrane.

T (K)	ΔG^0^ (kJ/mol)	ΔS^0^ (kJ/(mol∙K)	ΔH^0^ (kJ/mol)
298	−10.48	0.056	16.19
308	−13.96		
318	−18.19		

**Table 3:** Antifouling indexes of the membranes.

Membrane Types	R_t_	R_r_	R_ir_	FRR
PVDF	95.71	47.90	47.81	52.19
HFO-PVDF	94.39	84.14	10.25	89.75

## Data Availability

The original contributions presented in this study are included in the article. Further inquiries can be directed to the corresponding authors.
